# Psychotropic medication use in people living with severe and persistent mental illness in the Australian community: a cross-sectional study

**DOI:** 10.1186/s12888-022-04324-0

**Published:** 2022-11-15

**Authors:** Jie Hu, Sara S McMillan, Theo Theodoros, Jack C Collins, Sarira El-Den, Claire L O’Reilly, Amanda J Wheeler

**Affiliations:** 1 Menzies Health Institute Queensland, Griffith University, Brisbane, Australia; 2 School of Pharmacy and Medical Sciences, Griffith University, Brisbane, Australia; 3 Centre for Mental Health, Griffith University, Nathan campus, 4111 Brisbane, Australia; 4 University of Queensland Faculty of Medicine, Brisbane, Australia; 5 Metro South Mental Health Services, Brisbane, Australia; 6 Faculty of Medicine and Health, The University of Sydney School of Pharmacy, University of Sydney, Sydney, Australia; 7 Faculty of Medical and Health Sciences, University of Auckland, Auckland, New Zealand

**Keywords:** Psychotropics, Medication, Polypharmacy, Severe Mental illness

## Abstract

**Background:**

Psychotropic polypharmacy and high-dose prescribing may play a role in therapy, however, with associated risks. The aim of this study was to describe current prescribing practices and use of four psychotropic medication groups (antipsychotics, antidepressants, mood stabilisers and benzodiazepines), focusing on polypharmacy (across and within groups) and high-dose prescribing in adults experiencing severe and persistent mental illness (SPMI) in the Australian community.

**Methods:**

318 people taking psychotropic medication for SPMI had a medication review undertaken by a community pharmacist. Participants were recruited as part of an RCT from three Australian states/territories between September 2020-July 2021. All psychotropic medication and daily doses were recorded and reviewed for alignment with current clinical guidelines. Univariate and multiple logistic regression models investigated factors associated with antipsychotic, antidepressant, and mood stabiliser polypharmacy, and antipsychotic and antidepressant high-dose therapy. Variables included age, gender, geographic location, self- reported mental illness(es), hospital admission(s) in previous 6-months and prescriber type.

**Results:**

806 psychotropic medications were prescribed for the 318 participants. Mood stabiliser polypharmacy was recorded in 19.0% of participants prescribed mood stabilisers; antipsychotic polypharmacy in 18.4% of participants prescribed antipsychotics; antidepressant polypharmacy in 11.3% of those prescribed antidepressants; and three participants (5.1%) were prescribed two benzodiazepines concurrently. Almost 18.6% of the cohort was receiving high-dose treatment; 18 participants were prescribed high-dose antipsychotics and 39 high-dose antidepressants, with two participants prescribed both. Adjusted logistic regression for polypharmacy found male gender, psychiatrist as sole prescriber, or multiple prescribers, were associated with antipsychotic polypharmacy. The adjusted model for high-dose therapy found psychiatrist as sole prescriber was significantly associated with antipsychotic and antidepressant high-dose prescribing.

**Conclusion:**

Psychotropic polypharmacy was common in this community cohort experiencing SPMI. Whilst polypharmacy is not always inappropriate, it is a complex construct with potential benefits alongside potential risks. Benefits and harms need to be balanced however this practice is not supported by clear guidance to assist health practitioners. This study highlights the important need for regular medication reviews and strengthened communication between consumers and all healthcare professionals involved in community mental health care, to support safe and effective use of psychotropic medications.

## Introduction

Psychotropic medications, such as antipsychotics and mood stabilisers, are key elements in treating severe and persistent mental illness (SPMI), which was defined by the WHO as a group of conditions that include moderate to severe depression, bipolar disorder, schizophrenia and other psychotic disorders [1]. Balancing psychotropic medication effectiveness and safety for people living with SPMI, however, can be challenging [2]. Combined input from consumers, prescribers, pharmacists, and other healthcare professionals in treatment decision-making processes is thus essential in minimising medication burden and maximising benefits [3]. However, with the absence of consensus among existing clinical practice guidelines, particularly for complex and treatment-refractory illnesses, psychopharmacology prescriptions can vary based on diagnosis, treatment history and potential confounding comorbidities of the individual patient. Consumers may have limited involvement in treatment decisions for various reasons, such as insufficient medicine knowledge, impaired cognitive capacity, fragile mental health status, or if a therapeutic alliance has not been established with their relevant practitioners [4].

Polypharmacy refers to the co-prescribing of two or more psychotropic medications from either a different therapeutic group (e.g., an antipsychotic and an antidepressant) or from the same therapeutic group (e.g., two antipsychotics) [5]. A continuing concern relating to psychotropic polypharmacy is the conflicting evidence about effectiveness, and whether potential clinical benefits outweigh risks of possible adverse effects [5–7]. Whilst there may be specific indications when combining medications is justified (e.g. for someone experiencing co-occurring mental illnesses), prescribing the minimum effective amount of medication is considered good clinical practice as it reduces potential harm and promotes adherence to therapeutic regimens [8].

Concerns about psychotropic polypharmacy include unwanted and possibly harmful adverse effects [9–11], increased medication burden [5] and accumulated dose effects [12, 13]. A French study exploring the combination of antipsychotic and antidepressant use in a community-based sample found this combination was associated with more severe mental illness and higher mortality rates [14]. Amongst adults discharged from a secondary care mental health facility in London (England), antipsychotic polypharmacy was found to be associated with a higher risk of re-admission within six months in comparison to adults discharged on a single antipsychotic [15]. A subsequent English study, however, compared antipsychotic polypharmacy with monotherapy in a cohort of consumers living with SPMI and found it was not significantly related to increased risk of unplanned hospital admission, emergency department presentation, or death [16]. Finally, a recent review and meta-analysis of randomised controlled trials (RCTs) involving antipsychotic polypharmacy in people with schizophrenia suggested that there may be specific areas where this practice is beneficial, notably clozapine combined with aripiprazole [17].

Despite the equivocal research findings described above and clinical guidelines mostly recommending monotherapy, a high prevalence of psychotropic polypharmacy, particularly within the antipsychotic group, is reported in practice. For example, amongst a New Zealand sample of 2217 mental health consumers accessing community or hospital care the rate of antipsychotic polypharmacy was reported to be 26% [18]. In an Australian cohort of 800 people discharged from a mental health unit, varying rates of antipsychotic (33.8%), mood stabiliser (13.4%), antidepressant (9.5%) and benzodiazepine (3.6%) polypharmacy were observed [19]. A further cohort study using Australian pharmaceutical claiming records found the prevalence of antipsychotic polypharmacy and antidepressant polypharmacy had increased respectively from 5.9 to 7.3% and from 2.1 to 3.7% between 2006 and 2015 [20]. Increasing rates of antidepressant polypharmacy have also been reported internationally [21, 22].

Exploration of psychotropic polypharmacy both within and across therapeutic groups remains understudied in consumer populations living in community settings. Hence, the purpose of this paper is to describe current prescribing practices and usage of four psychotropic medication groups (antipsychotics, antidepressants, mood stabilisers and benzodiazepines), with a focus on polypharmacy (within and across therapeutic groups) and high-dose prescribing in adults living with SPMI in the Australian community. Important insights are anticipated for consumers and their carers, health professionals and policymakers.

## Methods

### Sample and setting

The study design adopted a cross-sectional observational approach and involved people living with SPMI in the Australian community. Participants were recruited into the Bridging the Gap between Physical and Mental Illness in Community Pharmacy Randomised Controlled Trial (*PharMIbridge* RCT) from three Australian states/territories, if they were aged 16 years or over, had used an antipsychotic or a mood stabiliser for SPMI continuously for the six months prior to recruitment, and had current medication problems and/or physical health concerns. All participants completed a medication review with a trained community pharmacist at baseline; the *PharMIbridge* RCT protocol is reported elsewhere [23]. A total of 318 participants were recruited between September 2020-July 2021 from Victoria, New South Wales, and the Australian Capital Territory, and are included in the analysis presented here.

Ethics approval was obtained from a University Human Research Ethics Committee (2019/473). All participants provided informed consent to participate in the RCT.

### Data collection

Pharmacists collected details about participants’ current mental health medications during an initial medication review. As part of the review process, medications were reconciled by the pharmacist from their current dispensing records and in conversation with the consumer. Medication review details were recorded in a cloud-based software platform (GuildCare NG^™^), which is currently used by many Australian community pharmacists when delivering professional services. Data included medication name, administration route, current dose and prescriber instructions.

Data on total polypharmacy across groups were calculated as the total aggregate of the four psychotropic medication groups for each participant. Within-group polypharmacy was calculated for the use of two or more psychotropic medications in the same group, specifically antipsychotics, antidepressants, mood stabilisers and benzodiazepines, including medications prescribed ‘if required (prn)’.

The process for determining high-dose therapy for antipsychotics, antidepressants and benzodiazepines has been previously described [19, 24]. For example, a total daily equivalent dose for each antipsychotic was calculated by the sum of the total daily dose divided by the recommended maximum daily dose. If more than one antipsychotic, antidepressant or benzodiazepine were prescribed, a cumulative total daily equivalent dose was calculated by summing the total daily equivalent dose scores for all relevant medications. High-dose therapy was defined as the total daily equivalent dose, or the cumulative total daily equivalent dose, being greater than one. Medications prescribed for prn use were excluded from the high dose analysis as were mood stabiliser because recommendations are lacking on a maximum therapeutic daily dose for lithium.

Participant demographic and clinical characteristics (including age, gender, country of birth, ethnicity, geographic location [urban or rural], history of hospitalisation in the previous 6-months, self-reported mental illness/es and current psychotropic medication prescriber(s) [GP, psychiatrist, hospital doctor, and/or nurse practitioner], were collected via a self- administered online survey.

### Statistical analyses

Data analyses were conducted using Stata Version 13.1 (StataCorp LP, USA). Participants’ demographic characteristics were reported as mean (SD, standard deviation) for continuous variables and frequency (percentage) for categorical variables. Descriptive analyses were conducted to examine the total across group polypharmacy and proportions of polypharmacy and high-dose therapy per psychotropic medication group. Univariable and multiple logistic regression models were used to investigate factors associated with antipsychotic, antidepressant, and mood stabiliser polypharmacy or antipsychotic and antidepressant high-dose therapy, respectively. Participant age, gender, geographic location, self-reported mental illness(es), hospital admission(s) in the previous six months and prescriber type were included in the multiple logistic regression models. Associated factors of polypharmacy or high-dose benzodiazepine prescribing were not examined due to the small sample size (n = 59) who were prescribed benzodiazepine treatment. Statistical difference was declared at *p* < 0.05.

## Results

### Participant characteristics

Table 1 outlines the demographic and clinical characteristics of the 318 participants included in this analysis; just over half were female (54.8%), the majority were born in Australia (91.5%). The mean age was 48.1 years (SD = 13.0 years; range = 18–82 years). According to the Modified Monash Model [25], which defines pharmacy location remoteness, over a third of participants were living in a non-metropolitan area (38.4%). More than half of participants (60.7%) self- reported living with more than one mental illness (median 2; range 1–5), predominantly moderate-severe mood and/or anxiety disorder(s), and just less than a quarter (23.3%) self-reported one or more hospital admissions in the six months immediately prior to recruitment (Table 1).

**Table 1:** Participant characteristics (n = 318)

Total population	n	%
Gender ^a^		
Male	140	45.2
Female	170	54.8
Age (years), mean (SD)	48.1 (13.0)	
18–29	27	8.5
30–44	102	32.1
45–59	139	43.7
60+	50	15.7
Country of birth		
Australia	291	91.5
Other		
Geographic residence	27	8.5
Metropolitan, MMM (1)	196	61.6
Non-metropolitan, MMM (2–7)	122	38.4
Self-reported hospital admissions (previous 6 months) *b*		
None	237	76.7
One	42	13.6
Two	17	5.5
Three	13	4.2
Self-reported mental illness *c*		
Depression (moderate/severe)	183	57.6
Anxiety disorder (moderate/severe)	142	44.7
Bipolar disorder	128	40.3
Schizophrenia/schizoaffective disorder	84	26.4
Personality disorder	38	12
Post traumatic stress disorder	33	10.4
Substance use	17	5.4
Self-reported prescriber for psychotropic medications		
GP only	148	46.5
Psychiatrist only	64	20.1
Multiple prescribers	99	31.1
Other ^*d*^	7	2.2

SD = Standard Deviation; IQR = Interquartile Range; MMM = Modified Monash Model; GP= General Practitioner

^a^not stated (n = 8)

^b^not stated (n = 9)

^c^participants could self-report more than one mental illness

^d^not stated (n = 6) or nurse practitioner (n = 1)

### Psychotropic medications prescribed

Details of the prescribed psychotropic medication groups are reported in Table 2. The average number of psychotropic medications prescribed per participant was two (range = 1–6) with most participants (83.3%, 265/318) prescribed medication across more than one psychotropic medication group; 11 (3.5%) participants were concomitantly prescribed medications from all four groups. The majority (83.6%, 266/318) of participants were prescribed one or more antipsychotics; 69.5% (221/318) were prescribed one or more antidepressants; 46.2% (147/318) were prescribed one or more mood stabilisers, and 18.5% (59/318) were prescribed one or two benzodiazepines. In total, 29.9% (95/318) were prescribed polypharmacy within one or more of the four medication groups.

**Table 2:** Psychotropic medication groups prescribed (n = 318)

Number of medications prescribed per individual	n	%
**Psychotropic group combinations**		
One psychotropic group	53	16.7
Two psychotropic groups	166	52.2
Three psychotropic groups	88	27.7
All four psychotropic groups	11	3.5
**Total number of psychotropic medications prescribed**		
One	39	12.3
Two	131	41.2
Three	102	32.1
Four	32	10.1
Five	13	4.1
Six	1	0.3
**Antipsychotic**		
None	52	16.4
One	217	68.2
Two	43	13.5
Three	6	1.9
**Mood stabiliser**		
None	171	53.8
One	119	37.4
Two	27	8.5
Three	1	0.3
**Antidepressant**		
None	97	30.5
One	196	61.6
Two	24	7.6
Three	1	0.3
**Benzodiazepine**		
None	259	81.5
One	56	17.6
Two	3	0.9

A total of 806 psychotropic medications were prescribed to the 318 participants. Table 3 outlines the prescription rates for the most commonly prescribed medications. Overall, quetiapine was the most commonly prescribed antipsychotic (37.4%), followed by olanzapine (25.9%) and aripiprazole (11.8%). Only 19 participants were prescribed a long-acting injectable (LAI) antipsychotic. Participants were prescribed a wide range of antidepressants (n = 20 medications), with venlafaxine being the most frequently prescribed (15.8%). Lithium (36.9%) and lamotrigine (36.4%) were the most commonly prescribed mood stabilisers. Diazepam accounted for 69.4% of the prescribed benzodiazepines.

**Table 3:** Prescription and high-dose rates of most frequently prescribed psychotropic medications (n = 806)

Prescribed medications	n (%)	High-dose (TDD/MDD > 1)
**Antipsychotics** Quetiapine Olanzapine Aripiprazole Clozapine Lurasidone Risperidone Paliperidone Zuclopenthixol Other	**321** 120 (37.4) 83 (25.9); (LAI n = 1) 38 (11.8); (LAI n = 6) 22 (6.9) 15 (4.7) 8 (2.5); (LAI n = 1) 7 (2.2); (LAI n = 6) 4 (1.3); (LAI n = 4) 24 (7.5); (LAI n = 1)	**8 (2.5)** 1 4 2 nil nil nil 1 nil nil
**Antidepressants** Venlafaxine Mirtazapine Sertraline Escitalopram Fluoxetine Desvenlafaxine Duloxetine Citalopram Nortriptyline Other	**247** 39 (15.8) 36 (14.6) 30 (12.2) 26 (10.5) 26 (10.5) 25 (10.1) 22 (8.9) 9 (3.7) 5 (2.0) 29 (11.7)	**25 (10.1)** nil 6 1 7 3 2 3 nil 1 2
**Benzodiazepines** Diazepam Lorazepam Clonazepam Temazepam Other	**62** 43 (69.4) 6 (9.7) 4 (6.5) 4 (6.5) 5 (8.1)	**nil**
**Mood stabilisers** Lithium Lamotrigine Valproate Topiramate Other	**176** 65 (36.9) 64 (36.4) 36 (20.5) 7 (4) 4 (2.3)	N/A*

LAI = long-acting injectable antipsychotic; TDD = total daily dose; MDD = maximum daily dose

*High dose information not available for mood stabilisers

### Polypharmacy within medication groups

Antipsychotic polypharmacy (two or more antipsychotics prescribed concurrently) was recorded in 18.4% (49/267) of participants prescribed antipsychotic treatment; antidepressant polypharmacy occurred in 11.3% (25/221) and mood stabiliser polypharmacy in 19.0% (28/147); only three participants (5.1%) were prescribed two benzodiazepines concurrently.

Of the 49 participants prescribed more than one antipsychotic, just over half were using olanzapine (n = 25) or quetiapine (n = 25); six participants were prescribed both medications. The other antipsychotic polypharmacy combination most frequently identified was quetiapine and aripiprazole (n = 6). Antipsychotic polypharmacy with clozapine plus aripiprazole was recorded in four participants, and clozapine plus amisulpride in one participant. Five out of seven participants who were prescribed oral paliperidone, had co-prescribed olanzapine and/or quetiapine. LAI antipsychotics were frequently co-prescribed with oral antipsychotics (57.9%; n = 11/19); three of these participants were co-prescribed two additional oral antipsychotics.

Of the 25 participants using more than one antidepressant, mirtazapine was most commonly prescribed (n = 18), followed by venlafaxine (n = 10). Among the 28 participants using more than one mood stabiliser, the most common combination was lithium with lamotrigine (64.3%; 18/28). Diazepam was co-prescribed with either clonazepam or temazepam in the three benzodiazepine polypharmacy observations.

### High-dose prescribing

Table 3 outlines the small number of single medication prescriptions that were identified as high-dose (total daily equivalent dose was greater than the recommended maximum daily dose), including 2.5% of antipsychotics and 10.1% of antidepressants.

A cumulative total daily equivalent dose was also calculated when more than one medication was prescribed within a psychotropic medication group. Overall, 18.6% (59/318) of the study population was receiving high-dose therapy, including 20 participants prescribed high-dose antipsychotic therapy and 41 high-dose antidepressant therapy, with two participants prescribed both. Specifically, fourteen of the 20 participants receiving high-dose antipsychotic therapy were prescribed antipsychotic polypharmacy and 21 of the 41 participants receiving high-dose antidepressant therapy were prescribed antidepressant polypharmacy.

### Variables associated with polypharmacy and high-dose prescribing

Logistic regression models explored the potential variables associated with antipsychotic, antidepressant, or mood stabiliser polypharmacy (Table 4), and antipsychotic or antidepressant high-dose prescribing (Table 5).

**Table 4:** Variables associated with polypharmacy

Variable	Antipsychotic		Antidepressant		Mood Stabiliser	
	**Crude OR (95% CI)**	^*a*^ **Adjusted OR (95% CI)**	**Crude OR (95% CI)**	**Adjusted OR (95% CI)**	**Crude OR (95% CI)**	**Adjusted OR (95% CI)**
Age	1.0* (0.9,1.0)	1.0 (1.0,1.0)	1.0 (1.0,1.1)	1.0* (1.0,1.1)	1.0 (1.0,1.0)	1.0 (1.0,1.0)
Gender Reference = Male	0.5* (0.3,1.0)	0.5* (0.2,1.0)	3.0* (1.1,8.2)	2.5 (0.9,7.3)	1.3 (0.5,3.1)	1.7 (0.6,4.6)
Geographic location Reference = Metropolitan	0.9 (0.5,1.6)	0.8 (0.4,1.7)	1.3 (0.6,3.0)	1.6 (0.6,3.9)	1.7 (0.7,3.9)	1.3 (0.5,3.3)
Mental illness Reference = 1 condition	1.5 (0.8,2.8)	1.4 (0.7,3.1)	1.6 (0.6,4.6)	2.2 (0.7,6.7)	0.4* (0.2,0.9)	0.6 (0.2,1.6)
Admission (last 6 months) Reference = Yes	1.3 (0.6,2.6)	1.0 (0.4,2.4)	1.7 (0.7,4.1)	1.9 (0.7,5.1)	0.7 (0.3,2.2)	0.7 (0.2,2.3)
Prescriber Reference = GP only Psychiatrist only ^ *b*^Multiple prescribers	1.0 (1.0,1.0) 5.3** (2.3,12.0) 2.8* (1.2,6.2)	1.0 (1.0,1.0) 6.2** (2.5,15.1) 3.0* (1.2,7.3)	1.0 (1.0,1.0) 2.1 (0.7,6.5) 1.9 (0.7,4.9)	1.0 (1.0,1.0) 2.3 (0.7,7.7) 2.0 (0.7,5.8)	1.0 (1.0,1.0) 2.1 (0.7,5.8) 0.6 (0.2,1.8)	1.0 (1.0,1.0) 2.08 (0.68,6.36) 0.57 (0.16,1.97)

*p< 0.05, **p< 0.001 ; OR, odds ratio; CI, confidence interval; GP, general practitioner

^a^All variables were included in the adjusted models

^b^Multiple prescribers consisted of GP and psychiatrist, GP and hospital doctor, or psychiatrist, hospital doctor and nurse practitioner

**Table 5:** Variables associated with high-dose prescribing

Variable	Antipsychotic		Antidepressant	
	**Crude OR** **(95% CI)**	^a^ **Adjusted OR** **(95% CI)**	**Crude OR** **(95% CI)**	**Adjusted OR** **(95% CI)**
Age	1.0 (0.9,1.0)	1.0 (0.9,1.0)	1.0 (1.0,1.0)	1.0 (1.0,1.1)
Gender Reference = Male	0.6 (0.2,1.5)	0.6 (0.2,1.6)	2.5* (1.1,5.4)	2.2 (1.0,4.9)
Geographic location Reference = Metropolitan	1.0 (0.4,2.6)	1.2 (0.5,3.3)	1.7 (0.8,3.3)	1.8 (0.9,3.8)
Mental illnesses Reference = one condition	1.3 (0.5,3.3)	1.3 (0.4,3.6)	0.8 (0.4,1.7)	0.9 (0.4,2.2)
Admission (last 6 months) Reference = Yes	0.8 (0.3,2.4)	0.7 (0.2,2.4)	1.5 (0.7,3.2)	1.3 (0.6,3.0)
Prescriber Reference = GP only	1.0 (1.0,1.0)	1.0 (1.0,1.0)	1.0 (1.0,1.0)	1.0 (1.0,1.0)
Psychiatrist only	4.6* (1.4,14.6)	4.5* (1.3,14.8)	2.6* (1.1,6.5)	2.7* (1.0,7.0)
^b^Multiple prescribers	2.5 (0.8,8.3)	1.9 (0.5,6.7)	1.7 (0.8,3.7)	1.6 (0.7,3.9)

*p< 0.05, **p< 0.001 ; OR, odds ratio; CI, confidence interval; GP, general practitioner

^a^All variables were included in the adjusted models

^b^Multiple prescribers consisted of GP and psychiatrist, GP and hospital doctor, or psychiatrist, hospital doctor and nurse practitioner

In the adjusted model for polypharmacy, only participant gender and prescriber type were found to be significantly associated with antipsychotic polypharmacy; male participants, those with a psychiatrist as their sole prescriber, or who received treatment from multiple prescribers (GP and psychiatrist, GP and hospital doctor, or psychiatrist, hospital doctor and nurse prescriber), were more likely to be prescribed multiple antipsychotics.

In the adjusted model for high-dose treatment, only having a psychiatrist as the sole prescriber was significantly associated with antipsychotic and antidepressant high-dose prescribing.

## Discussion

This study examined psychotropic medication prescriptions, and the prevalence of polypharmacy and/or high-dose therapies of these medications in a sample of people experiencing SPMI living in the Australian community. Overall, most participants had been prescribed more than one psychotropic medication and from more than one psychotropic medication group. The total proportion of participants receiving polypharmacy therapy within any of the four medication groups was 29.9%; the highest rates were observed for antipsychotics and mood stabilisers. In total, less than 20% of participants were receiving high-dose treatment.

Quetiapine was the most commonly prescribed antipsychotic, which has broad licensed indications in Australia for the treatment of schizophrenia, bipolar affective disorder, major depressive disorders and generalised anxiety disorders. The greater prescription of venlafaxine and mirtazapine antidepressants in this study is also not unexpected due to their balance of efficacy and tolerability and are recommended treatment for depression in the Royal Australian and New Zealand College of Psychiatrists (RANZCP) clinical practice guidelines for mood disorders [26]. The use of diazepam as the most common benzodiazepine in this cohort is of potential concern requiring further investigation, given its long half-life and current recommendations for benzodiazepines to be prescribed for short-term use [27].

The high proportion of participants using more than one psychotropic medication (83.3%) emphasises treatment complexity. Given that participants were living with a SPMI and 61% of them self-reported experiencing more than one mental illness, the proportion of polypharmacy and high-dose treatment observed within each psychotropic group was not unexpected. Clinical practice guidelines do not endorse high-dose therapy due to limited evidence on benefits or potential adverse effects [28]. Whilst clinical practice guidelines (in Australia and elsewhere) provide limited advice on polypharmacy, the literature suggests that combinations of more than one antipsychotic, antidepressant or mood stabiliser may be used by specialist mental health practitioners for short-term individual use, providing symptom response and adverse effects are closely monitored [8, 28]. Recommendations are provided for longer-term polytherapy trials for people experiencing a lack of therapeutic response or poor outcomes with monotherapy after adequate trials, e.g. clozapine combined with aripiprazole for treatment-refractory schizophrenia [17]. Co-prescription of lithium and lamotrigine, as the use of alternate mood-stabiliser polypharmacy, is also supported by clinical practice guidelines for bipolar affective disorders [26].

However, two controversial practices with an increased risk of harm identified in this study were: 1) the high rate of antipsychotic polypharmacy involving a LAI, where 57.9% of this sub-group were co-prescribed another oral antipsychotic and 15.8% were prescribed another two; and ii) antipsychotic combinations most commonly involved sedating antipsychotics quetiapine or olanzapine.

With respect to the first practice, co-prescribing an LAI with an additional oral antipsychotic may have a clinical rationale including: providing appropriate antipsychotic coverage when switching from oral to LAI treatment (i.e. until steady-state is reached, particularly with risperidone and aripiprazole); supplementation for acute periods of breakthrough psychotic symptoms; when a dose increase of the LAI is being pursued; and augmenting the effects of one antipsychotic (particularly in situations involving a partial response to a clozapine trial [17]). The use of two oral antipsychotics in addition to the LAI is however more challenging to rationalise. Whilst it may reflect prescribing during a period of acute and severe illness, justifying long-term co-prescription of three antipsychotics is difficult given current evidence and clinical practice guidelines.

Higher prescribing rates of quetiapine and olanzapine in this cohort are likely to be a reflection of the self-reported high prevalence of mood disorders in this cohort. Typically, however it is considered preferable to select an appropriate antipsychotic, mood stabiliser or antidepressant for long-term use and only use additional sedative or anxiolytic treatment short-term or if required. Whilst this may explain the rationale for the co-prescribing of sedating antipsychotics observed in this cross-sectional study, it highlights the need to be mindful of the physical impacts of antipsychotics on morbidity and mortality and the need for regular medication reviews.

Whilst the observed rates of antipsychotic polypharmacy (18.4%) in this community-based study population were similar to global reports of a median prevalence of 19.6% [29], they are lower than another Australian point of discharge study which reported 27.4% antipsychotic polypharmacy amongst consumers discharged from an inpatient unit [19]. Similarly, our community-based study observed high-dose antipsychotic treatment rates of only 6.2% compared to 24.4% in the aforementioned point of discharge study. Both of these findings indicate alignment with current Australian clinical practice guidelines for the treatment of schizophrenia and related disorders [30], and international guidelines such as the Maudsley Prescribing Guidelines [31].

In contrast, the polypharmacy rates of mood stabilisers, antidepressants and benzodiazepines reported in our study (19.0%, 11.3%, and 5.1% respectively) were considerably higher than the rates observed in the point of discharge study (3.4%, 3.9%, and 1.0% respectively); however high-dose treatment rates with antidepressants and benzodiazepines were similar across the two studies [19]. Likely, these two studies may have ultimately included consumers with quite different diagnosis, with this current study including a greater number of people with affective disorders and the discharge cohort experiencing more psychotic disorders. Unfortunately, by way of contextualising these findings, we were unable to locate other comparable studies that described psychotropic polypharmacy and high-dose therapy more widely than antipsychotics. Whilst polypharmacy was not common practice (rates all less than 20%), it is important to highlight the risks associated with using multiple medications and the need to schedule regular reviews of polypharmacy regimens to ensure they are providing ongoing therapeutic benefit and minimal adverse effects.

Our current study adds to the existing knowledge about the influence of the prescribing environment in the context of psychotropic polypharmacy and high-dose therapy. In this community-based study, participants were six times more likely to be prescribed antipsychotic polypharmacy and four times more likely to be prescribed high-dose therapy when a psychiatrist was the sole prescriber. Additionally, participants were two and a half times more likely to be prescribed high-dose antidepressant therapy when a psychiatrist was the sole prescriber. These findings are reassuring to suggest that the more complex psychopharmacology prescription is occurring with specialised prescribers (psychiatrists) supporting general practitioners. Despite challenges accessing psychiatrists in Australia [32], people with complex presentations were engaging with specialist services. However, this research should highlight the ongoing need for such specialised input.

Conversely, this study also found an independent association when multiple prescribers were involved; participants were almost three times more likely to be prescribed antipsychotic polypharmacy when compared to those who only reported having a GP involved in their prescribing. Brett et al. examined psychotropic polypharmacy Australia-wide using Pharmaceutical Benefits Scheme (PBS) claims data and found over a quarter (26.7%) of all polypharmacy episodes involved multiple prescribers [20]. These authors raised concerns about poor communication and potential dilution of responsibility between multiple prescribers for regular monitoring of the medication regimen prescribed (including polytherapy) and adverse effects. Similarly, Kouladjian et al. found that situations involving a GP and a specialist co-prescribing were a barrier to de-prescribing of sedatives and anticholinergic medications [33].

Further medication management challenges may exist when multiple prescribers are involved. Another Australian study examining the shared care for clozapine consumers, which involved a psychiatrist providing a prescription of clozapine supply, a GP undertaking mandatory monitoring, and a hospital pharmacy dispensing and delivering clozapine to community pharmacy or GP for consumer collection, found high rates of discrepancies in the medication records held by each prescriber and the pharmacies supplying the medications [34]. Consumers involved in this program also identified issues and confusion about communication mechanisms and roles within this multiple prescriber arrangement [35]. Consequently, all healthcare professionals have an important role in medication reconciliation and communication of information, including empowering consumers to play an active role in these pathways to improve medication outcomes [36]. The impact of the community pharmacist in reviewing the medication use and supporting this study population will be a focus of future analysis from the *PharMIbridge* RCT, thereby providing important insight into any changes in psychotropic polypharmacy and high dose prescribing practices.

Compared to studies using electronic health and dispensing records, or administrative datasets [20, 37], our study used a novel approach with data collected through medication reviews, during which pharmacists obtained, verified, and documented a list of current medications with each study participant. Reconciling medications via such a formal process has been previously described [38]. The study findings uniquely reflect the actual medication burden of consumers living with an SPMI in the Australian community.

However, our study results should be interpreted with respect to limitations. Firstly, psychotropic medications that were used for conditions other than mental illness, such as insomnia and epilepsy, were excluded (n = 65) from the analysis. We also excluded other psychotropic medications which were outside the four aforementioned groups (e.g. disulfiram, medications for attention deficit hyperactivity disorder), although we acknowledge that the use of other psychotropic medications may contribute significantly to overall medication burden and risk of adverse effects. Therefore, polypharmacy and/or high-dose prescribing practices could be underestimated. Participant demographic and clinical information, including prescriber information for psychotropic medications, was collected through a self-administered questionnaire, which may result in possible information bias. There may be a selection bias as participants had self-identified and agreed to participate in a RCT evaluating the impact of a pharmacist intervention on physical and mental well-being. Results need to be interpreted with respect to the RCT selection criteria as participants were likely to have used at least one antipsychotic or mood stabiliser. Additionally, the selection of the covariates in the multiple logistic regression was based on reported factors from the literature; potential confounding factors such as primary diagnosis, years of mental illnesses, the severity of symptoms etc. were not included. Many participants reported multiple and co-existing mental illnesses; associations between specific mental illnesses and the risk of polypharmacy and/or high-dose prescribing were not tested. Instead, multiple mental illnesses were included as a covariate in the logistic regression models. Lastly, although the study endeavoured to cover a range of pharmacy types (e.g. based on locality, size and population served) in three Australian states, the results may not be generalisable elsewhere, including beyond Australia. This study provides important insights, however, into psychotropic medication prescribing and use in a community-based population living with SPMI. Consumers as well as prescribers’ experiences with, and perceptions of, prescribing, including possible benefits and side effects of their psychotropic medications, were not explored in this study, which should shed light on future endeavors for achieving optimal psychotropic treatment for people living with SPMI in the community.

## Conclusion

Overall, psychotropic polypharmacy was common in a community cohort of people living with SPMI. Whilst polypharmacy is not always inappropriate, it is a complex construct with potential benefits and potential risks. This study did not identify a greater divergence in clinical practice with respect to polypharmacy and high-dose prescribing compared to published literature. Whilst some identified trends were concerning, such as the use of three concurrent antipsychotics, a higher use of quetiapine and olanzapine, particularly as part of antipsychotic polypharmacy, they highlight the importance of regular medication reviews and strengthened communications between consumers, and all healthcare professionals involved in community mental health care.

## Data Availability

The data that support the findings of this study are available upon request but restrictions apply to the availability of these data, which are not publicly available. Please contact the corresponding author for relevant request.
